# Comparative Predictive Performance of Serum and Dose-Normalized Valproate Levels for Seizure Control in Adults With Epilepsy

**DOI:** 10.7759/cureus.108869

**Published:** 2026-05-14

**Authors:** Govind Mishra, Hina Lal, Sneha Ambwani

**Affiliations:** 1 Pharmacology, Dr. Ram Manohar Lohia Institute of Medical Sciences, Lucknow, IND; 2 Pharmacology, Maharishi Markandeshwar Institute of Medical Sciences and Research, Ambala, IND; 3 Pharmacology, All India Institute of Medical Sciences (AIIMS), Jodhpur, IND

**Keywords:** epilepsy, receiver operating characteristic (roc) curve, seizure control, therapeutic drug monitoring, valproate

## Abstract

Background

Valproate is a widely used antiepileptic drug, but its clinical response shows considerable interindividual variability. Conventional therapeutic ranges may not reliably predict seizure control, and the role of dose-normalized valproate levels in forecasting seizure as an outcome remains unclear.

Objective

To evaluate the predictive performance of serum valproate levels for seizure control and compare it with dose-normalized valproate levels, and also to assess whether multivariable models incorporating demographic factors improve the prediction of seizures in adult patients with epilepsy.

Methods

This study was a secondary analysis of prospectively collected data from adult patients with epilepsy receiving valproate monotherapy at a tertiary care centre. Demographic and clinical data were recorded, and patients were followed for one month to assess seizure occurrence. Serum valproate levels and dose-normalized levels were analysed at the end of the follow-up period. Logistic regression models were constructed, and predictive performance was assessed using ROC analysis.

Results

A total of 137 patients were included, of whom 30 (21.9%) experienced seizures during follow-up. Serum valproate levels demonstrated good predictive performance for seizure control (AUC: 0.891, 95% CI: 0.814-0.969), with an optimal cutoff of 56.1 µg/mL (sensitivity 86.7%, specificity 86.9%). Dose-normalized levels showed lower predictive performance (AUC: 0.829, 95% CI: 0.734-0.923). Logistic regression analysis confirmed serum valproate level as a significant predictor of seizure control, while dose-normalized levels showed weaker associations. Model-based predictions demonstrated comparable performance but did not significantly outperform serum levels.

Conclusion

Serum valproate levels provide a clinically meaningful prediction of seizure control and outperform dose-normalized metrics. These findings may support the use of concentration-based monitoring to guide individualized therapy in epilepsy.

## Introduction

Epilepsy is a disorder of brain function that accounts for a significant proportion of the disease burden globally, characterized by the periodic and unprovoked occurrence of seizures [1]. Anti-seizure medications remain the cornerstone of epilepsy management and exert their therapeutic effects by modulating neuronal excitability. Despite advances in antiepileptic therapy, achieving optimal seizure control remains a major clinical challenge, with a substantial proportion of patients continuing to experience breakthrough seizures [2]. Effective management, therefore, requires not only appropriate drug selection but also individualized dosing strategies to maximize efficacy while minimizing adverse effects [3].

Valproate is widely used for broad and effective coverage of different types of seizure activity. However, its use is complicated by a tight therapeutic range and marked inter-individual variability in its pharmacokinetics [4]. Plasma concentrations of valproic acid (VPA) are often correlated with administered dose; their relationship with therapeutic response remains inconsistent, limiting the clinical utility of fixed therapeutic ranges, although a therapeutic plasma concentration range of 50-100 µg/mL is recommended [5,6].

An alternative approach to account for variability in dosing is the use of dose-normalized drug levels, which adjust serum concentration relative to administered dose per body weight. In theory, such normalization may better reflect individual pharmacokinetic differences and provide a more individualized measure of drug exposure [7]. However, dose-normalized valproate levels have been proposed to account for variability in dosing relative to body weight and interindividual differences in exposure [8]. While this approach may help account for variability related to dose intensity and body weight, its relationship with seizure outcomes has not been well characterized, and comparative data with directly measured serum concentrations remain limited.

Furthermore, while statistical models incorporating multiple patient characteristics may improve predictive accuracy, it is uncertain whether these models provide meaningful advantages over simple, clinically accessible biomarkers such as serum drug concentration [9,10]. Clarifying the relative predictive performance of serum valproate levels, dose-normalized levels, and multivariable models is therefore of practical importance for optimizing therapeutic drug monitoring (TDM) strategies in routine clinical care. The present study was therefore undertaken to evaluate the predictive performance of serum valproate levels for seizure control in adult patients with epilepsy and to assess the clinical utility of dose-normalized valproate levels as an alternative marker of drug exposure. Additionally, the study aimed to compare the discriminatory performance of these parameters for seizure prediction.

## Materials and methods

Study setting

This study is a secondary analysis of the pre-approved prospective observational study. The data was originally collected from the Department of Neurology at All India Institute of Medical Sciences (AIIMS), Jodhpur, a tertiary care teaching hospital, from March 2018 to October 2019. The present analysis was subsequently performed in the Department of Pharmacology at Dr. Ram Manohar Lohia Institute of Medical Sciences (Dr. RMLIMS), Lucknow. The study protocol was approved by the Institutional Ethics Committee of AIIMS Jodhpur (Approval No: AIIMS/IEC/2018/440). The present study was conducted on a de-identified patient data collected under the approved protocol. Prior to analysis, all direct patient identifiers were removed, and anonymized study codes were used to maintain confidentiality throughout the study.

Study population

Adult patients aged 18-60 years with a diagnosis of epilepsy attending the neurology outpatient department were screened for eligibility. Patients receiving valproate monotherapy and providing written informed consent were included. Patients receiving concomitant antiepileptic drugs, those with known hepatic or renal impairment, and those unwilling to participate were excluded.

Data collection

Baseline demographic and clinical data were recorded at enrolment using a structured data collection form. These included age, body weight, and treatment-related variables such as total daily valproate dose (mg) and dose per kilogram body weight (mg/kg). A derived variable, the dose-normalized valproate level, was calculated as the ratio of serum valproate concentration (µg/mL) to dose per kilogram (mg/kg) and expressed as µg/mL per mg/kg [7]. All data was anonymized prior to analysis using a coded dataset and removing any identifiers. Patients were followed up after one month as per the original data collection protocol, and seizure occurrence during the follow-up period was assessed based on the patient-maintained seizure diary. The diary contained details of the drug taken and the presence or absence of seizures on a daily basis. Seizure outcome was categorized as a binary variable: presence of seizure (uncontrolled) or absence of seizure (controlled). Blood samples were collected after one month of therapy to measure steady-state trough plasma valproate concentrations. Plasma was separated by centrifugation and stored at −80°C until analysis. Valproate levels were quantified using high-performance liquid chromatography (HPLC) [11]. Patients were maintained on stable valproate dosing during the follow-up period, and no dose adjustments were made prior to blood sampling. Serum valproate concentrations were measured under steady-state conditions, immediately prior to the next scheduled dose. The study involved minimal risk to participants due to its observational design, and blood sample collection was performed by trained personnel using standard institutional procedures.

Sample size calculation

The sample size for the original study was calculated based on its primary objective. The present analysis represents a secondary analysis of the available dataset. All eligible patients with complete data during the study period were included in the final analysis, resulting in a sample size of 137 participants.

Statistical analysis

Statistical analysis was performed using R statistical software version 4.5.0 (R Foundation for Statistical Computing, Vienna, Austria). Continuous variables were summarized as mean ± standard deviation, while categorical variables were expressed as frequencies and percentages. Univariate logistic regression analysis was conducted to evaluate the association between serum valproate level, dose-normalized valproate level and seizure occurrence. Multivariable logistic regression models were then constructed to identify independent predictors of seizure outcome: Model 1 included serum valproate level, age, and body weight, while Model 2 included dose-normalized valproate level, age, and body weight. Results were reported as odds ratios with 95% confidence intervals.

Receiver operating characteristic (ROC) curve analysis was performed to assess the discriminatory ability of serum valproate level/dose-normalized valproate level, and predicted probabilities derived from the multivariable models. The area under the ROC curve (AUC) with 95% confidence intervals was calculated using the DeLong method [12,13]. The optimal cut-off was selected using the Youden index, and sensitivity, specificity, and accuracy were reported [14]. Comparative analysis of ROC curves was performed using DeLong’s test for correlated ROC curves. Model performance was further evaluated using the Akaike Information Criterion (AIC), with lower values indicating better model fit [15]. Only cases with complete data for all variables were included in the final analysis.

## Results

A total of 137 patients receiving valproate therapy were included in the final analysis after exclusion of incomplete records. Baseline demographic and clinical characteristics are presented in Table 1.

**Table 1:** Baseline characteristics of the study population *Data is presented as Mean ± SD and frequency (%).

Characteristic	Descriptive statistic * (N=137)
Age (years)	30.6 ± 11.3
Weight (kg)	65.1 ± 11.1
Valproate dose (mg)	804 ± 252.3
Dose per kg (mg/kg)	12.6 ± 4.3
Serum valproate level (µg/mL)	84.5 ± 45.0
Dose-normalized valproate level (µg/mL per mg/kg)	6.69 ± 3.25
Seizure present, n (%)	30 (21.9)
Seizure absent, n (%)	107 (78.1)

The mean age of the study population was 30.6 ± 11.3 years, and the mean body weight was 65.1 ± 11.1 kg. The mean serum valproate level was 84.5 ± 45.0 µg/mL, while the mean dose-normalized valproate level was 6.69 ± 3.25 µg/mL per mg/kg. Seizure occurrence was documented in 30 (21.9%) patients, whereas 107 (78.1%) patients were seizure-free.

Serum valproate level and seizure outcome

In univariate regression analysis, serum valproate level was significantly associated with seizure occurrence. Each unit increase in serum valproate level was associated with a reduction in the odds of seizure (OR 0.95, 95% CI: 0.93-0.97, p < 0.001). A similar result was observed in the multivariable analysis of serum valproate level with seizure occurrence as well. Each unit increase in serum valproate level was associated with a reduction in the odds of seizure (adjusted OR 0.944, 95% CI: 0.921-0.961, p < 0.001). Among covariates, body weight was significantly associated with increased seizure risk (p = 0.007), while age did not show a statistically significant association (p = 0.155). Detailed regression results are shown in Table 2.

**Table 2:** Multivariable analysis of serum and dose-normalized valproate levels. *p < 0.05 is considered statistically significant.

Model	Variable	Adjusted OR (95% CI)	p-value
Model 1: Serum valproate level-based model	Serum valproate level	0.944 (0.921–0.961)	<0.001*
	Age	1.036 (0.987–1.089)	0.155
	Weight	1.076 (1.023–1.141)	0.007*
Model 2: Dose-normalized valproate level-based model	Dose-normalized valproate level	0.499 (0.372–0.634)	<0.001*
	Age	1.025 (0.981–1.070)	0.260
	Weight	1.106 (1.049–1.177)	<0.001*

**Figure 1:**
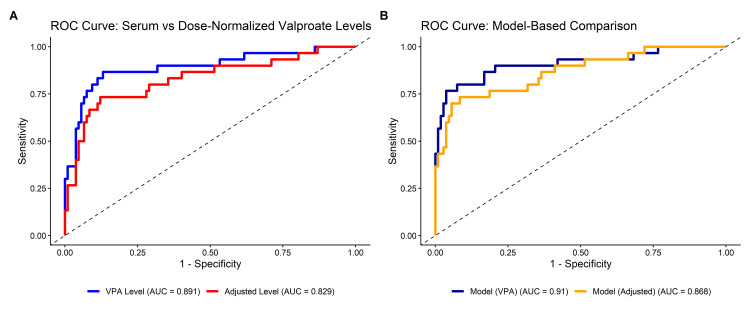
Receiver operating characteristic (ROC) curves comparing predictive performance of valproate-based metrics. (A) Serum valproate level versus dose-normalized valproate level for prediction of seizure outcome. (B) Multivariable logistic regression models based on serum valproate level and dose-normalized valproate level.

Receiver operating characteristic (ROC) analysis demonstrated that serum valproate level had good discriminatory ability for predicting seizure outcomes, with an area under the curve (AUC) of 0.891 (95% CI: 0.814-0.969). The optimal cut-off value was 56.1 µg/mL, corresponding to a sensitivity of 86.7%, specificity of 86.9%, and overall accuracy of 86.9% (Table 3). The comparative ROC curves are shown in Figure 1A, and the ROC curve for the model is shown in Figure 1B. A multivariable logistic regression model incorporating serum valproate level, age, and body weight demonstrated slightly improved predictive performance, with an AUC of 0.910 (95% CI: 0.823-0.982). The optimal predicted probability threshold was 0.487, yielding a sensitivity of 76.7%, specificity of 96.3%, and accuracy of 92.0% (Table 3).

**Table 3:** Diagnostic performance of valproate-based predictors for seizure outcome

Parameter	AUC (95% CI)	Cut-off	Sensitivity (%)	Specificity (%)	Accuracy (%)
Serum valproate level	0.891 (0.814–0.969)	56.06	86.7	87.9	87.6
Dose-normalized valproate level	0.829 (0.734–0.923)	4.62	73.3	87.9	84.7
Model 1: Serum valproate level-based model	0.91 (0.838–0.982)	0.487	76.7	96.3	92.0
Model 2: Dose-normalized valproate level-based model	0.868 (0.788–0.947)	0.31	73.3	91.6	87.6

Dose-normalised valproate level and seizure outcome

Dose-normalized valproate level was also significantly associated with seizure occurrence, with lower levels associated with higher seizure risk (OR 0.60, 95% CI: 0.48-0.73, p < 0.001). It was also independently associated with seizure occurrence in multivariable analysis. Each unit increase in dose-normalized level was associated with a reduction in seizure risk (adjusted OR 0.499, 95% CI: 0.372-0.634, p < 0.001). Body weight remained significantly associated with seizure occurrence (p = 0.001), while age was not statistically significant (p = 0.260). These results are detailed in Table 2. ROC analysis for dose-normalized valproate level demonstrated lower discriminatory performance compared to serum valproate level, with an AUC of 0.829 (95% CI: 0.734-0.923). The optimal cut-off value was 4.62, yielding a sensitivity of 73.3%, specificity of 87.9%, and accuracy of 84.7% (Table 3).

Comparative analysis of predictive performance

Direct comparison of ROC curves demonstrated that serum valproate level had significantly superior discriminatory performance compared to dose-normalized valproate level (AUC 0.891 vs 0.829, p = 0.028) (Table 3, Figure 1A). Similarly, the multivariable model based on serum valproate level showed higher AUC compared to the model based on dose-normalized levels (0.910 vs 0.868); however, this difference was not statistically significant (p = 0.123) (Figure 1B). Additionally, model fit was superior for the serum valproate level-based model, as indicated by a lower Akaike Information Criterion (AIC 85.3 vs 98.5).

Overall, serum valproate level demonstrated strong predictive ability for seizure control, outperforming dose-normalized valproate levels. Multivariable adjustment provided only a marginal improvement in predictive performance.

## Discussion

In this study, serum valproate levels exhibited better performance compared to dose-normalized metrics and good predictive ability for seizure control.

Literature search revealed that many previous studies have focused on clinical and treatment-related factors rather than quantitative drug exposure while evaluating seizure outcomes in patients on valproate. For instance, a retrospective cohort study conducted by Gandelman-Marton and Theitler through review of the computerized database and the medical records of 10 years in valproate-treated epileptic adults reported that seizure outcomes were influenced by clinical factors such as gender and treatment patterns, with varying associations among treatment characteristics and seizure control [16]. Similarly, Prabhakaran et al. conducted a randomized controlled study to compare valproate with other antiseizure medications by evaluating seizure control/reduction rates and clinical efficacy without integrating serum drug concentrations as predictive markers [17]. Even in landmark comparative trials by Mattson et al., effectiveness in achieving seizure control and other outcomes was assessed based on clinical endpoints such as seizure frequency and remission, rather than individualized pharmacokinetic drug exposure [18]. Together, these findings highlight that the role of measurable drug exposure parameters in predicting clinical outcomes has been relatively underexplored despite valproate being a cornerstone in epilepsy management. This gap emphasizes the significance of the current study, which methodically evaluates serum drug levels as a predictor of seizure control using a quantitative and discriminative approach.

Substantial variability in valproate, supported by pharmacokinetic evidence, has been demonstrated in the current study. Precision therapeutic drug monitoring data report by Landmark et al. demonstrated unpredictable exposure even under stable dosing, significant intra- and inter-individual variability for both total and unbound concentrations, with coefficients of variation approaching 55% [19]. Population pharmacokinetic studies further validate that valproate clearance is influenced by various patient-specific factors such as age, body weight, sex, daily dose, and concomitant medications. Thus, substantial variability in serum concentrations with comparable dosing is observed [20,21]. Earlier clinical pharmacokinetic studies have similarly demonstrated considerable interindividual variability in the relationship between administered valproate dose and achieved serum concentrations during monotherapy. Tisdale et al. reported only a moderate correlation between valproate dose and serum concentration despite stable dosing conditions, suggesting that administered dose alone may not reliably reflect systemic exposure [22]. Nonlinear and saturable protein binding leads to disproportionate increases in the pharmacologically active unbound fraction at higher concentrations, further increasing the variability [19]. In addition, additional studies indicate that a significant proportion of patients fail to achieve target therapeutic levels in routine practice due to variability in dosing and drug interactions [23]. Serum valproate levels, as a direct measure of systemic exposure, demonstrated superior discriminatory performance in relation to seizure occurrence in the present study. Collectively, these findings provide pharmacokinetic and clinical context supporting the observed association between measured serum valproate concentrations and seizure outcomes.

In contrast to total serum valproate levels, dose-normalized valproate concentrations did not demonstrate comparable predictive performance for seizure control in the present study. This can be attributed to the complex and multifactorial pharmacokinetics of valproate. The concentration-to-dose (C/D) ratio is influenced by numerous patient-specific and biological variables and does not reflect a stable exposure metric. Wu et al. demonstrated that normalized valproate concentrations are significantly affected by genetic polymorphisms in metabolic enzymes such as uridine diphosphate glucuronosyltransferase (UGT) isoforms. These alleles can alter drug clearance independent of the administered dose [24]. Additionally, demographic and clinical factors including age, sex, body weight, and comedication have been demonstrated to significantly modify dose-adjusted concentrations through their impact on valproate metabolism and distribution [9]. Due to nonlinear protein binding, there is a higher free fraction at higher concentrations. Thus, discordance between total concentration, free drug exposure, and dose-normalized indices is seen in valproate [25,26]. Dose normalization may oversimplify the underlying pharmacokinetic variability and can cause failure in capturing the true pharmacologically active exposure. These factors likely explain why dose-normalized levels in the present study did not correlate as strongly with seizure outcomes, reinforcing the limitation of relying on normalized metrics in clinical practice.

The findings of the present study should be interpreted in the context of evolving predictive approaches in epilepsy. Although TDM is widely utilized, evidence suggests that regular monitoring for predefined ranges does not consistently improve outcomes [25]. Xing et al. used an approach using biomarker-based and machine learning models, such as Lasso-logistic regression incorporating genes like SNORD3A, to demonstrate moderate predictive performance (AUC ~0.70) [27]. However, such models require advanced techniques such as molecular profiling, which are not readily applicable in routine clinical settings. In contrast, our findings demonstrate that simple serum valproate levels provide clinically meaningful prediction of seizure control, supporting a pragmatic, exposure-based approach for individualized therapy.

Limitations

This study has several limitations. The single-center design, modest sample size, and short follow-up duration may limit generalizability. In addition, several clinically relevant variables, including seizure type, duration of epilepsy, baseline seizure frequency, adherence patterns, and concomitant non-antiepileptic medications, were not systematically available for inclusion in the multivariable analysis. Therefore, residual confounding cannot be excluded, and the observed associations should be interpreted cautiously, as the observed associations may partly reflect underlying disease severity or treatment-related factors. The predictive performance estimates and ROC-derived cut-offs should be interpreted cautiously, as the same dataset was used for both model development and performance evaluation. Furthermore, internal validation methods were not performed, and the findings therefore require validation in independent cohorts before clinical application.

## Conclusions

Serum valproate concentrations demonstrated superior discriminatory performance compared with dose-normalized valproate levels in relation to seizure occurrence in adult patients with epilepsy. Directly measured serum concentrations appeared to better reflect clinically relevant drug exposure than derived dose-normalized metrics. However, the identified serum valproate cut-off and model performance estimates should be interpreted cautiously, as these findings were derived from a single-center exploratory dataset without external validation. Keeping in mind the exploratory nature of the study, further prospective studies are required to validate these observations and determine their clinical applicability.
